# Molecular Scoring of Hepatocellular Carcinoma for Predicting Metastatic Recurrence and Requirements of Systemic Chemotherapy

**DOI:** 10.3390/cancers10100367

**Published:** 2018-09-29

**Authors:** Naoshi Nishida, Takafumi Nishimura, Toshimi Kaido, Kosuke Minaga, Kentaro Yamao, Ken Kamata, Mamoru Takenaka, Hiroshi Ida, Satoru Hagiwara, Yasunori Minami, Toshiharu Sakurai, Tomohiro Watanabe, Masatoshi Kudo

**Affiliations:** 1 Department of Gastroenterology and Hepatology, Faculty of Medicine, Kindai University, 377-2 Ohno-higashi, Osaka-sayama, Osaka 589-8511, Japan; kousukeminaga@yahoo.co.jp (K.M.); yamaken_volvo@yahoo.co.jp (K.Y.); ky11@leto.eonet.ne.jp (K.K.); matakenaka@med.kindai.ac.jp (M.T.); hidakuhp@gmail.com (H.I.); hagi-318@hotmail.co.jp (S.H.); minkunminami@yahoo.co.jp (Y.M.); sakurai@med.kindai.ac.jp (T.S.); tomohiro@med.kindai.ac.jp (T.W.); m-kudo@med.kindai.ac.jp (M.K.); 2 Department of Medical Oncology, Kitano Hospital, 2-4-20 Ohgi-machi, Kita-ku, Oaska 530-8511, Japan; nisimura@kuhp.kyoto-u.ac.jp; 3 Division Hepato-Biliary-Pancreatic Surgery and Transplantation, Department of Surgery, Kyoto University Graduate School of Medicine, 54 Karahara-cho, Syogoin, Sakyo-ku, Kyoto 606-8507, Japan; kaido@kuhp.kyoto-u.ac.jp

**Keywords:** hepatocellular carcinoma, recurrence, molecular subclass, mutation, methylation, chromosomal alteration, liver transplantation, systemic chemotherapy

## Abstract

Hepatocellular carcinoma (HCC) causes one of the most frequent cancer-related deaths; an HCC subset shows rapid progression that affects survival. We clarify molecular features of aggressive HCC, and establish a molecular scoring system that predicts metastasis after curative treatment. In total, 125 HCCs were examined for *TP53*, *CTNNB1*, and *TERT* promoter mutation, methylation of 8 tumor suppressor genes, and 3 repetitive DNA sequences to estimate promoter hypermethylation and global hypomethylation. A fractional allelic loss (FAL) was calculated to represent chromosomal instability through microsatellite analysis. Molecular subclasses were determined using corresponding and hierarchical clustering analyses. Next, twenty-five HCC patients who underwent liver transplantation were analyzed for associations between molecular characteristics and metastatic recurrence; survival analyses were validated using a publicly available dataset of 376 HCC cases from the Cancer Genome Atlas (TCGA). An HCC subtype characterized by *TP53* mutation, high FAL, and global hypomethylation was associated with aggressive tumor characteristics, like vascular invasion; *CTNNB1* mutation was a feature of the less-progressive phenotype. A number of molecular risk factors, including *TP53* mutation, high FAL, significant global hypomethylation, and absence of *CTNNB1* mutation, were noted to predict shorter recurrence-free survival in patients who underwent liver transplantation (*p* = 0.0090 by log-rank test). These findings were validated in a cohort of resected HCC cases from TCGA (*p* = 0.0076). We concluded that molecular risks determined by common genetic and epigenetic alterations could predict metastatic recurrence after curative treatments, and could be a marker for considering systemic therapy for HCC patients.

## 1. Introduction

Hepatocellular carcinoma (HCC) is the sixth most common cancer and the third most frequent cause of cancer-related death worldwide. Receiving curative treatment, such as radiofrequency ablation, hepatic resection, and liver transplantation, is critical for the long survival of HCC patients [1]. However, emergence of metastatic recurrence after curative treatment has a strong negative impact on survival [2]. Therefore, identifying a subset of HCC that carries a high risk of metastasis is clinically important, because patients with tumor dissemination need to be treated in combination with systemic therapy.

So far, sorafenib has been the only agent effective for advanced stage HCCs, including those with metastatic recurrence [3]. However, in addition to sorafenib, several molecular targeting agents have been found to be effective for the treatment of advanced HCC. For example, regorafenib, a tyrosine kinase inhibitor (TKI) targeting vascular endothelial growth factor receptor (VEGFR) type 1 to 3, platelet-derived growth factor receptor, angiopoietin 2 receptor, KIT, and RET, is now approved for HCC patients who have progressed on sorafenib, because of its effect on extension of overall survival (OS) and progression-free survival (PFS) [4]. This result suggests that sequential sorafenib/regorafenib treatment would be available in the advanced stage of HCCs, which could be beneficial for survival [5]. Another multikinase inhibitor, lenvatinib, has also been approved for HCC cases refractory to locoregional therapies [6]. Furthermore, the anti-VEGFR2 antibody, ramucirumab, is reportedly beneficial to HCC patients with serum alpha-fetoprotein (AFP) levels ≥400 ng/dL, and cabozantinib, that mainly inhibits tyrosine kinases including MET, AXL, VEGFR-2, is also known to induce objective tumor response with reduction of tumor markers [7,8]. In addition, anti-programmed cell death 1 (PD-1) antibody is reportedly show an objective response in around 20% of HCC cases [9,10,11]. Considering that these agents showed promising results for patients with disseminated HCC, treatment strategies and management of progressed HCC should be reconsidered because locoregional treatment sometimes result in deterioration of liver function [5]. Currently, systemic therapies of HCC are recommended for the cases with advanced stage where portal vein invasion and extrahepatic spread are detected [12]. However, HCC patients who need systemic therapies should be selected more precisely, because undetectable tumor dissemination causes metastatic recurrence after curative treatment; new systemic therapies, using TKIs and immune checkpoint inhibitors, could improve survival in such HCC cases.

On the other hand, cancer cells carry several genetic, epigenetic, and chromosomal alterations; these events could have a strong impact on acquisition of aggressive tumor behavior [13]. For example, accumulation of chromosomal alterations, as well as genome-wide DNA hypomethylation, is reportedly associated with HCC aggressiveness [14,15,16]. A recent genome-wide mutational analysis also showed driver mutations on several cancer-related genes associated with HCC progression [17,18]. In addition, inactivation of tumor suppressor genes (TSGs) by promoter methylation is also critical for HCC emergence and progression [19,20,21,22,23]. Despite observations that HCCs harbor several kinds of alterations that may affect tumor behavior, a majority of studies merely focus on one of these alterations to predict tumor characteristics. However, as these events are generally observed simultaneously, thus indicating a crosstalk among cancer-related pathways, it is important to consider comprehensive aspects of molecular events to determine the tumor characteristics [24,25,26]. From this perspective, we conducted comprehensive genetic, epigenetic, and chromosomal analyses in the same HCC tissues, and subclassified them to determine molecular characteristics related to tumor aggressiveness. In addition, this study also aimed at assessing the performance of molecular risk scoring, constructed through molecular subclassification, on the emergence of metastatic recurrence after curative therapies, like liver transplantation in HCC patients. Based on these data, we speculate molecular risk score can be a good marker for the selection of HCC patients who need systemic therapies with novel TKIs and immune checkpoint inhibitors.

## 2. Results

### 2.1. Mutations of CTNNB1 and TP53 Genes and TERT Promoter, in HCCs from Hepatic Resection

In total, 24.8% (31/125), 21.6% (27/125), and 68.0% (85/125) of HCCs showed mutation in *CTNNB1* and *TP53* genes and the *TERT* promoter, respectively. Details of the position and base substitution of each mutation are listed in Supplementary Table S1. We analyzed the association between the presence of mutation and clinical background: sex (male or female), age (>60 or ≤60 years), presence of HBV (hepatitis B surface antigen; HBsAg positive or negative), presence of HCV (hepatitis C antibody; HCVAb positive or negative), serum AFP level (≥200 ng/mL or <200 ng/mL), maximum tumor size (≥3.0 cm or <3.0 cm), vascular invasion (presence or absence), tumor number (solitary or multiple), and differentiation of tumor (well or moderately/poorly).

Mutation of *CTNNB1* was more frequently observed in HCCs from males (28/90, 31.1% for males, and 3/35, 8.6% for females; *p* = 0.0088 by Pearson’s chi-square test, Table 1). Similarly, *CTNNB1* was associated with low serum AFP levels <200 ng/mL (*p* = 0.0015), absence of vascular invasion (*p* = 0.0194), and a well-differentiated phenotype (*p* = 0.0018). Mutation of the *TP53* gene was associated with HBV-positivity (*p* = 0.0147), serum AFP levels ≥200 ng/mL (*p* = 0.0063), presence of vascular invasion (*p* = 0.0029), and moderately/poorly-differentiated phenotype (*p* = 0.0235). On the contrary, the clinical backgrounds associated with *TERT* promoter mutation was the presence of HCV and without non-B non-C (NBNC) (*p* = 0.0007 and 0.0282 for HCV and NBNC, Table 1).

### 2.2. Regional Hypermethylation at TSG Promoters and Global Hypomethylation in HCCs from Hepatic Resection

Median methylation levels and 25th–75th percentiles on the promoters of 8 TSGs, determined by combined bisulfite restriction analyses (COBRA), are as follows: 52.0% (distribution, 36.5–70.0), 23.0% (0–45.8), 39.0% (19.0–62.0), 29.7% (5.1–46.7), 42.0% (11.6–74.0), 9.0% (0–26.9), 38.4% (0–58.5), and 13.9% (0–34.3) for the promoters of *APC*, *CDKN2A*, *RASSF1A*, *HIC-1*, *GSTP1*, *RUNX3*, *SOCS1*, and *PRDM2*, respectively. Similarly, the median methylation levels and 25th–75th percentiles on repetitive DNA sequences (rDNAs) determined by MethyLight are as follows; 31.8% (23.3–43.7), 28.0% (16.3–43.5), and 34.9 (14.5–65.5) for *Alu*, long interspersed element-1 (*LINE-1*), and juxtacentromeric satellite 2 (*SAT2*), respectively. Based on the methylation levels of 8 TSG promoters, and 3 kinds of rDNAs, we determined the presence of regional TSG hypermethylation and significant global hypomethylation, respectively, using hierarchical analyses (Supplementary Figure S1). Eighty-one HCCs were classified as carrying TSG hypermethylation, whereas 44 were without hypermethylation. Similarly, among 125 HCCs, 67 were considered as carrying significant global hypomethylation.

The presence of TSG hypermethylation showed a borderline association with presence of HCV (*p* = 0.0494). On the contrary, tumors showing global hypomethylation were significantly more frequent in patients with serum AFP levels ≥200 ng/mL, and with vascular invasion (*p* = 0.0469 and 0.0216, respectively, Table 2). Although not statistically significant, the global hypomethylation phenotype also showed borderline association with the presence of HBV and absence of HCV (*p* = 0.0583 and 0.0519, respectively, Table 2).

### 2.3. Degree of Chromosomal Alterations in HCC

The median fractional allelic loss (FAL) score of the HCC cohort was 21% (distribution; 2–64); we classified HCCs based on the FAL score as an alternative of total extension of chromosomal alteration; 67 HCCs showed FAL scores ≥ 21%, and 58 HCCs showed FAL scores < 21%. High FAL scores were significantly associated with high serum AFP levels (*p* < 0.0001), presence of vascular invasion (*p* = 0.0043), and moderately/poorly differentiated phenotype (*p* = 0.0393, Table 2).

### 2.4. Molecular Classification of HCC Based on Comprehensive Analyses of Mutation, DNA Methylation, and Chromosomal Alterations

**Figure 1:**
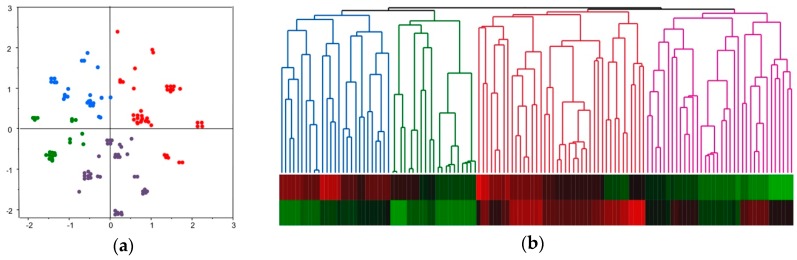
Molecular classification of HCC based on corresponding and hierarchical clustering analyses. Members of the A1-subclass are shown in blue, A2-subclass in green, B1-subclass in red, and B2-subclass in purple. (**a**) 125 HCCs were analyzed using the corresponding analysis based on the presence or absence or the *CTNNB1*, *TP53*, and *TERT* promoter mutations, methylation status on 8 TSG promoters (with or without hypermethylation), methylation status on the 3 kinds of rDNAs (with or without significant hypomethylation), and FAL score (<21% and ≥21%). (**b**) Hierarchical clustering analyses using x- and y-axis values of two-dimensional drawings of corresponding analysis shown in (**a**). Each subclass was determined based on the clusters.

Each HCC showed several alterations in cancer-related genes and chromosomes, and they might act in concert and affect the establishment of aggressive tumor phenotypes. Therefore, we attempted to consider each molecular alteration comprehensively for molecular classification and examined the clinical characteristics of each subclass. For this purpose, we performed corresponding analyses and visualized the association of each sample based on molecular alterations (Figure 1a). Subsequently, we classified HCCs using hierarchical clustering.

**Figure 2:**
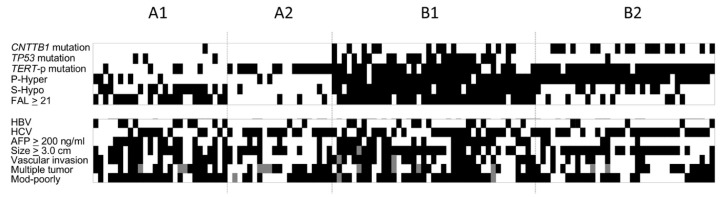
Heat-map of the molecular alterations and clinical background of the cases in each subclass. The black rectangle represents positive, the white represents absence, and gray shows that information is missing. *p*-hyper denotes promoter hypermethylation determined by methylation levels of 8 tumor suppressor genes, and S-hypo denotes significant global hypomethylation determined by methylation levels of 3 kinds of repetitive DNA sequences. *TERT*-p mutation, *TERT* promoter mutation. FAL: fractional allelic loss (%) as a representative of the degree of chromosomal alterations. Mod-poorly: moderately-poorly differentiated.

One hundred and twenty-five HCCs were successfully classified into 4 groups through hierarchical clustering (Figure 1b). The proportion of subclasses A1, A2, B1, B2 were 21.6% (27/125), 16.8% (21/125), 32.8% (41/125), and 28.8% (36/125), respectively. An overview of genetic, epigenetic, and chromosomal alterations of each subclass is shown in Figure 2. Briefly, *CTNNB1* mutations were detected in 3.7% (1/27), 0% (0/21), 31.7% (13/41), and 47.2% (17/36) of the tumors in subgroups A1, A2, B1, and B2, respectively. Similarly, the frequencies of *TP53* and *TERT* promoter mutations in each subgroup were as follows: 11.1% (3/27), 0% (0/21), 56.1% (23/41), and 2.8% (1/36) for *TP53* mutation, and 18.5% (5/27), 71.4% (15/21), 75.6% (31/41), and 94.4% (34/36) for *TERT* promoter mutations in subgroup A1, A2, B1, and B2, respectively (*p* < 0.0001 for *CTNNB1*, *TP53*, and *TERT* promoter mutation; Table 3). Regarding methylation status, 22.2% (6/27), 9.5% (2/21), 95.1% (39/41), and 94.4% (34/36) of the tumors showed hypermethylation in TSG promoters, and 59.3% (16/27), 9.5% (2/21), 92.7% (38/41), and 30.6% (11/36) represented significant global hypomethylation in subgroups A1, A2, B1, and B2, respectively (*p* < 0.0001 for both; Table 3). For extension of altered chromosomal regions, 70.4% (19/27), 14.3% (3/21), 90.2% (37/41), and 22.2% (8/36) showed FAL scores ≥ 21% (*p* < 0.0001; Table 3).

Based on these analyses, group A was characterized by low frequencies of *CTNNB1* and *TP53* mutation, and TSG hypermethylation; the significant global hypomethylation and FAL ≥ 21% were more frequent in subgroup A1 than in A2. On the other hand, high frequency of *CTNNB1* mutation and TSG hypermethylation were characteristic of group B. In addition, the frequencies of *TP53* mutation, global hypomethylation, and FAL ≥ 21% were the highest in subgroup B1 among the 4 subgroups.

### 2.5. Molecular Classification and Clinical Feature of HCC

Next, we examined the differences in clinical factors associated with tumor aggressiveness among the molecular subgroups. There was no difference in age, sex, tumor size, and number of tumors among groups; though statistically insignificant, the proportion of HBV positivity was higher in subgroup A1 and B1 than in A2 and B2. HCV positive patients differed among the groups where HCV related HCC was more frequent in subgroups A2 and B2 than in A1 (*p* = 0.0149; Table 3). Interestingly, there was a clear difference in the proportion of factors related to tumor aggressiveness and metastasis, such as high serum AFP levels, vascular invasion (*p* = 0.0002 for serum AFP levels and *p* = 0.0013 for vascular invasion). Subgroup B1 showed the highest proportion of tumors with serum AFP ≥ 200 ng/mL and positive for vascular invasion (56.1% and 64.1%, respectively), which was followed by subgroup A1 (51.9% and 63.0% for high serum AFP and vascular invasion, respectively; Table 3). Moderately/poorly differentiated phenotype was also more frequent in the HCCs from subgroup A1 and B1, compared to the tumors in subgroup A2 and B2.

We also compared recurrence-free survival (RFS) after the initial treatment among the patients classified into 4 molecular subgroups. Interestingly, patients in subgroup A1 and B1 showed shorter RFS than those in A2 and B2 (*p* = 0.0222; Supplementary Figure S2).

### 2.6. Scoring Using Molecular Risk Factors and Recurrence after Liver Transplantation in HCC

Next, we also scored HCC cases of liver transplantation based on the number of molecular alterations. Details of patients who underwent liver transplantation are shown in Supplementary Table S2. Among the 25 patients, HCC recurrence emerged in 6 patients during the median observation period of 50 months (range 2–106 months); all these patients showed extrahepatic recurrence.

Among patients who underwent transplantation, 7 (28%: 7/25), 9 (36%: 9/25), and 18 (72%: 18/25) showed *CTNNB1*, *TP53*, and *TERT* promoter mutations, respectively (Supplementary Table S3); 37.5% of the patients (9/25) showed FAL scores ≥ 21%. Median methylation levels and 25th–75th percentiles on the promoters of 8 TSGs in this sample cohort are as follows: 44.8% (distribution; 28.1–66.8), 23.0% (0–52.3), 71.1% (47.3–90.2), 45.7% (11.1–70.7), 27.6% (0–43.0), 4.4% (0–16.8), 38.0% (25.3–52.9), and 11.3% (3.9–20.6) for the promoters of *APC*, *CDKN2A*, *RASSF1A*, *HIC-1*, *GSTP1*, *RUNX3*, *SOCS1*, and *PRDM2*, respectively. Similarly, median methylation levels and 25th–75th percentiles on rDNAs are as follows: 64.0% (57.5–74.8), 46.5% (25.3–70.7), and 38.5% (10.5–68.5) for *Alu*, *LINE-1*, and *SAT2*, respectively.

Based on the analysis using liver resection cases, the molecular classification of HCC reflected the tumor characteristics well, where the aggressive subgroups were characterized by the presence of *TP53* mutation, FAL scores ≥ 21%, and significant global hypomethylation (Table 3). Absence of *CTNNB1* mutation is also related to high serum AFP levels and vascular invasion (Table 1). Therefore, we scored HCCs from liver transplantation cases using the total number of following items: absence of *CTNNB1* mutation, presence of *TP53* mutations, FAL score ≥ 21%, and global hypomethylation phenotype; we arbitrarily subclassified HCCs as showing an “aggressive molecular pattern” if the number of items ≥3, and as “mild molecular pattern” if the number of items ≤2.

For determination of significant global hypomethylation in 25 HCCs from liver transplantation, we used the *Z*-score of methylation levels in 125 HCCs from liver resection, which was calculated using the mean and standard deviation of methylation levels of *Alu*, *LINE-1*, and *SAT2* as follows: mean methylation level = 0.36, standard deviation = 0.25, *Z*-score = (“methylation levels of each cases” − 0.36)/0.25. Subsequently, we calculated the sum of the *Z* scores of *Alu*, *LINE-1*, and *SAT2*. A receiver operating characteristic (ROC) curve revealed the sum of *Z*-scores, “−0.01217,” as the best threshold to discriminate significant global hypomethylation from non-hypomethylation phenotype in the HCC cohort of liver resection. Therefore, we considered HCCs from liver transplantation as carrying global hypomethylation phenotype if the sum of *Z*-scores < −0.01217.

**Figure 3:**
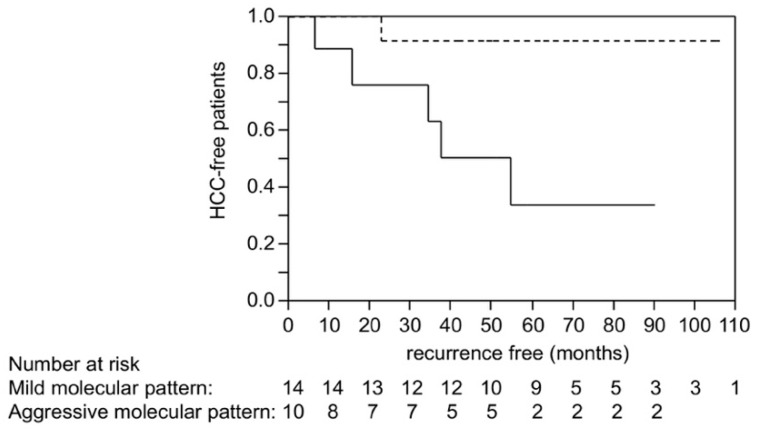
Recurrence-free survival of HCC patients who underwent liver transplantation. The solid line represents the survival of cases with the aggressive molecular pattern (molecular risk factors ≥ 3), and the broken line represents the cases with mild molecular pattern (molecular risk factors ≤ 2). *p* = 0.0090 by log-rank test.

We then compared RFS after liver transplantation between HCC patients showing aggressive molecular patterns and those showing mild molecular patterns. The RFS of patients with HCCs showing aggressive molecular pattern was significantly shorter than those showing mild molecular pattern (*p* = 0.0090 by log-rank test; Figure 3). Serum AFP and decarboxy-prothrombin (DCP) levels and tumor size were also significantly associated with the DFS in univariate analysis (Supplementary Table S4). We subsequently performed multivariate analysis considering serum AFP and decarboxy-prothrombin (DCP) levels, tumor size, and molecular pattern; no items were revealed to be independent (Supplementary Table S4).

### 2.7. Survival Analysis Using Dataset from TCGA

**Figure 4:**
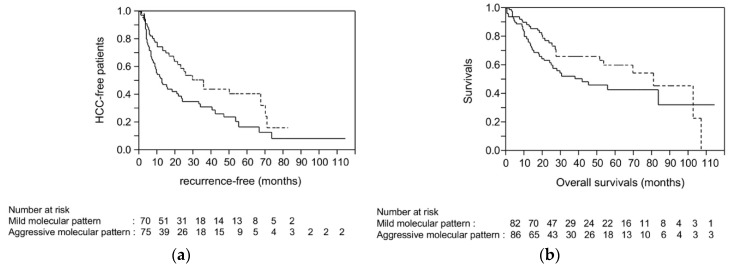
Recurrence-free survival (**a**) and overall survival (**b**) of HCC patients who underwent liver resection. The dataset included 376 HCCs referred from the Cancer Genome Atlas (TCGA). Among these, the results of whole exome sequencing, copy number values by Affymetrix SNP6, methylation analysis by HumanMethylation450 BeadChip, and clinical data, including the survival and curability of resection, are available for 168 HCC cases. These were subjected to Kaplan-Meier analysis. Since genome-wide methylation analysis was not applicable, the number of molecular risk factors ≥2 was considered as an aggressive molecular pattern, and those with 0–1 molecular risk factor was considered as showing a mild molecular pattern. The solid and the broken lines represent the survival of cases with aggressive and mild molecular patterns, respectively. (**a**) Kaplan–Meier curve for recurrence-free survival; *p* = 0.0076 by log-rank test. (**b**) Kaplan–Meier curve for overall survival; *p* = 0.1037 by log-rank test.

For validation of the robustness of the molecular risks for recurrence determined by our HCC cohort, we referred to the TCGA database where a clinical dataset of 376 HCC cases are available. Among them, data from whole exome sequencing, copy number values determined using Affymetrix SNP6, and methylation analysis by HumanMethylation450 BeadChip (HM450) are available for 168 HCC cases. The details of the 168-HCC dataset are shown in Supplementary Table S5. As the methylation levels of rDNAs are not available in this dataset, we scored HCC cases based on three molecular risk factors: absence of *CTNNB1* mutation, presence of *TP53* mutation, and high FAL score. For determination of FAL, copy number gain and loss were analyzed in 24,776 genes. The mean and median percentage of copy number alteration was 40% and 37% of this cohort, respectively. Therefore, HCCs with percentage of copy number alteration ≥39% were considered as showing high FAL. The RFS after HCC resection was significantly shorter in HCC cases with multiple molecular risk factors (2 or 3 risk factors) compared to those with 0–1 risk factor (*p* = 0.0076 by log-rank test; Figure 4a). Although not significant, overall survival was also shorter in HCC cases with multiple molecular risk factors (*p* = 0.1037; Figure 4b). We also performed survival analysis using 152 HCC cases with curative resection (no cancer cells observed microscopically at the resection margin, denoted as R0 in Supplementary Table S5) among 168 HCC cases. Again, the RFS was significantly shorter in HCC cases with multiple molecular risks than in those with 0–1 molecular risk factor (*p* = 0.0058).

## 3. Discussion

A variety of alterations in cancer-related genes have been detected in human cancers. For example, mutations in oncogenes and TSGs, loss and gain of chromosomal regions, regional methylation in the TSG promoters, and global hypomethylation, are recurrently reported in cancers including HCC [13,26]. These alterations were reportedly associated with characteristics unique to cancer such as cell proliferation, invasion, and metastasis; it is conceivable that such alterations could act in concert and contribute to the establishment of aggressive tumor behavior. However, because of the complexity of genetic and epigenetic alterations that are observed in a single tumor, it is difficult to classify HCC based on molecular events. In this study, we extensively analyzed the molecular alterations of HCC. Based on these findings, we tried to classify HCC, and found that the classification and scoring system using common molecular alterations was beneficial to select the cases with high risk of tumor metastasis by dissemination after curative treatment.

For mutational analyses, we determined mutations in *CTNNB1* and *TP53* genes and in the *TERT* promoter that are commonly detected in HCC. So far, the effect of *CTNNB1* mutation on tumor behavior is controversial. Several reports have showed that the presence of *CTNNB1* mutation is associated with less aggressive tumor phenotypes, whereas the *TP53* mutation represents HCC progression [27,28,29]. On the other hand, another study reported no association between *CTNNB1* mutation and patient survival [30]. Rebouissou et al. showed that altered β-catenin activity was attributed to the position of the affected amino acid, where a cancer-specific phenotype was associated with mutations within the beta-transducin repeat-containing protein (β-Trcp) binding site [30]. The majority of *CTNNB1* mutations detected our analysis were within the β-Trcp binding site, although some showed mutation at codon 41 and 45, that represented moderate and weak β-catenin activity, respectively. Recently, it was also reported that a non-proliferative class of HCC characterized by well-differentiated and low-aggressive phenotype consists of two subclasses characterized by gene expression patterns similar to perivenous and periportal hepatocytes, denoted as perivenous (PV) type and periportal (PP) type, respectively; the former shows frequent *CTNNB1* mutation and the latter is associated with wild-type *CTNNB1* [31]. Although the PP-type of HCC with wild-type *CTNNB1* is characterized by low risk of metastatic recurrence, tumors with wild-type *CTNNB1* also include proliferative type of HCC, such as those showing cancer extracellular matrix (ECM) remodeling/epithelial mesenchymal transition and stem cell (STEM) phenotype. Based on our classification using 125 HCC samples, the A2-subclass showed no *CTNNB1* mutation, with the lowest rate of *TP53* mutation, global hypomethylation, and a high FAL index. This subclass was characterized by low serum AFP levels, absence of vascular invasion, and a well-differentiated phenotype. Therefore, the PP-type of HCC, described by Desert et al., might roughly match the A2-subclass of our classification [31]. However, a considerable number of HCCs with wild-type *CTNNB1* were members of the A1- and B1-subclass with *TP53* mutation, global hypomethylation, chromosomal instability, and aggressive tumor phenotype, which could be characteristics of ECM- and STEM-type HCC. Therefore, the HCC cases with *CTNNB1* mutation in our cohort generally showed a relatively less aggressive phenotype, compared to those with wild-type *CTNNB1*, although some cases with wild-*CTNNB1* might also be less aggressive.

On the other hand, *TP53* mutation has been reported to be associated with tumor progression [28]. We have previously shown that *TP53* mutation was a character of advanced tumors, and was associated with the presence of chromosomal instability and global hypomethylation [16]. Boyault et al. also reported that *TP53* mutation was frequently accompanied by chromosomal instability phenotype [32]. Hoshida et al. proposed that absence of *CTNNB1* and presence of *TP53* mutation is a characteristic for aggressive HCCs positive for stem cell markers such as cytokeratin 19 and Encamp [27]. Our data also demonstrated that A2- and B2-subtypes were less aggressive, whereas A1 and B1 were more related to the aggressive phenotype; *CTNNB1* mutation was most frequent in B2, and *TP53* mutation, FAL ≥ 21%, and significant global hypomethylation were characteristics of A1- and B1-subtypes. Therefore, it is conceivable that the combined presence of *TP53* mutation, chromosomal instability, and global hypomethylation, might have a negative impact on the survival of HCC patients.

Although *TERT* promoter mutation and hypermethylation in TSG promoters were frequent in both B1 and B2, these were also commonly detected in other subtypes of HCC. Previously, we reported that hypermethylation of the *APC*, *CDKN2A*, *RASSF1A*, *HIC-1*, *GSTP1*, *RUNX3*, *SOCS1*, and *PRDM2* genes is a frequent event, even in early HCC [33], suggesting that hypermethylation of these TSGs could play a role in the early stage of hepatocarcinogenesis, and is independent of metastatic potential acquisition. *TERT* promoter mutation is also reportedly detected in preneoplastic lesions as well as at the early stage of HCCs [34]. Our analysis showed that the presence of *TERT* promoter mutation and hypermethylation in TSG promoters were associated with HCV presence, but not with characteristics related to tumor progression (Table 1). Therefore, the methylation status of 8 selected TSGs and the *TERT* promoter should be a common feature of hepatocarcinogenesis, but may not necessarily reflect an aggressive tumor phenotype.

So far, many studies have been focused on the molecular factors of HCC that could affect the prognosis of the patients. For example, Chiang et al. analyzed copy number alteration and clinical outcomes among the patients who underwent surgical resection; gains of chromosome 7 were the risk of recurrence after surgical resection [35]. However, as the cases of recurrence after liver resection involve metastatic recurrence, as well as multicentric occurrence of de novo HCC that can be attributed to the carcinogenic potential of liver cirrhosis, it is unclear whether the molecular change was definitely associated with tumor metastasis or not. To clarify the molecular risk related to the metastatic recurrence, we then focused on the analysis of the cases with liver transplantation, where all recurrences are metastatic. Through the analysis of a large number of HCC and non-cancerous liver tissues obtained from liver resection, we considered that the molecular risk factors for tumor metastasis were the presence of *TP53* mutation, broad chromosomal alteration, and global hypomethylation accompanied by the absence of *CTNNB1* mutation. With these four molecular events, we established a scoring system and confirmed its performance using an HCC cohort with liver transplantation. For evaluating this scoring system, we specifically selected liver transplantation cases, because, as described above, the recurrences after the transplantation should not involve multicentric occurrence of de novo HCC. As shown in Figure 3, HCC cases with 3 or more molecular risk factors (aggressive molecular pattern) showed shorter RFS compared to those with molecular risk factors ≤2 (mild molecular pattern). Due to the lack of the number of the patients who underwent liver transplantation, we also validated the performance of the molecular scoring system using the HCC dataset from TCGA, and successfully found that the molecular risk scores were also significantly associated with recurrence after curative liver resection. Through analysis using these strict validation cohorts, we found that scoring based on the commonly detected molecular events predict recurrence after curative treatment of HCC.

In this study, we classified HCCs based on commonly detected molecular events in tumors. As mentioned above, the genetic and epigenetic alterations in each HCC are complex, and should be affected by each other. This complicity makes the establishment of molecular-based HCC classification and a scoring system difficult to accomplish in a logical manner. From this perspective, we successfully established a molecular scoring system that reflects metastatic recurrence after curative treatment of HCC. Despite our comprehensive molecular analysis and robust validation, there are several limitations to this study; we analyzed the mutation and methylation of selected genes, which are commonly reported in HCC. Recent advancement of sequencing technology has revealed many genetic and epigenetic changes in HCC, although the majority of these alterations are not frequent enough to apply in molecular scoring systems [36,37]. Therefore, the role of minor alterations reported in HCC on survival remains to be clarified. In addition, it is also unclear whether molecular alterations detected with low clonality might affect the biological characteristics of the tumor, which is associated with the patient fate. Nevertheless, based on the robustness of the validation in this analysis, we proposed that combined molecular analyses targeting common alterations in HCC are important in clinical practice; HCC patients in early/intermediate stage with high molecular risks can be treated by locoregional therapy or resection, in combination with systemic therapies, such as TKIs and immune-checkpoint inhibitors.

## 4. Materials and Methods

### 4.1. Patients

In total, 125 HCC and their non-cancerous liver tissues were obtained from liver resections, and analyzed for the classification of HCC tissues based on gene mutations, promoter methylation of TSGs, genome-wide hypomethylation, and chromosomal alterations. The details of the clinical background of the patients are shown in Supplementary Table S6. Briefly, 95 patients were male and 35 were female. Median age was 63 years old (25th–75th percentile; 56–69). Twenty-seven patients are positive for HBsAg, 75 were positive for HCVAb, two were positive for both, and 21 were negative for both. The median maximum size was 3.6 cm (25th–75th percentile; 2.7–6). Fifty-eight and 65 HCCs were with and without vascular invasion, respectively (two were missing). Fifty-five were solitary, 58 were multiple (12 were missing). Thirty-six tumors were well-differentiated, 64 were moderately and 21 were poorly differentiated tumor (4 are missing). We used archived tissue samples for this study; the samples were obtained with the consent of patients at Kyoto University Hospital between April 1992 and April 2007.

For analyzing the molecular score of HCC based on comprehensive molecular analysis and emergence of metastatic recurrence, we examined HCC patients who underwent liver transplantation, because all recurrences after transplantation should be metastatic. In total, 25 patients were analyzed, and 6 showed recurrence during the median follow-up period of 30 months (range, 106.2–2.1); all the recurrences were extrahepatic. The details of the clinical background of the patients are shown in Supplementary Table S2. Liver transplantations were performed between April 2004 and November 2009 at Kyoto University Hospital. Both HCCs and their noncancerous tissues obtained during liver resection and liver transplantation were stored at −80 °C until DNA extraction. Association between the molecular score and survival after curative treatment was also validated in a dataset consisting of 376 HCCs from TCGA. The results of whole exome sequencing, copy number values determined by Affymetrix SNP6, methylation analysis by HM450, and clinical data, were downloaded from the TCGA web site in September 2017 (data source project ID; TCGA-LIHC). This study was approved by the ethics committee of the Kindai University Hospital (25-216 on 17 June 2014) and the Kyoto University Hospital (G679 on 13 November 2014). The study protocol conforms to the ethical guidelines of the 1975 Declaration of Helsinki.

### 4.2. Mutational Analysis of Cancer-Related Genes in HCC

We analyzed mutations of *CTNNB1* and *TP53* genes and the *TERT* promoter, because these are the most frequently mutated genes of HCC. Exon 3 of *CTNNB1* and exons 5–8 of *TP53* were analyzed for mutations in both HCC and non-cancerous liver tissues. Somatic mutations were confirmed by sequencing both sense and antisense strands. The *TERT* promoter was also analyzed, including the mutational hot spots at −124 and −146 bp from the ATG start site. Sequencing was performed using the direct Sanger technique. The details of the PCR primers and conditions were reported previously, and are summarized in Supplementary Table S7 [24,28,38].

### 4.3. Detection of Regional Promoter Methylation of Tumor Suppressor Genes in HCC

We analyzed methylation levels in the promoter region of *APC*, *CDKN2A*, *RASSF1A*, *HIC-1*, *GSTP1*, *RUNX3*, *SOCS1*, and *PRDM2*, because these promoters showed frequent and dense methylation in HCC tissues compared to non-cancerous liver tissues, and an inverse relation was observed between methylation levels and gene expression through our previous analyses. For quantification of methylation levels, we applied COBRA. The details of the primers and restriction enzymes for COBRA were reported previously [33]. Bisulfite-treated CpGenome Universal Methylated DNA (CHEMICON International Inc., Temecula, CA, USA) was used as a positive control for methylated samples.

### 4.4. Detection of Global Hypomethylation in HCC

We quantified methylation levels of three types of rDNAs, *LINE-1*, *Alu*, and *SAT2* because these methylation levels reportedly reflect the degree of global hypomethylation. Methylation levels were quantified using the MethyLight assay (StepOne real-time detection system; Applied Biosystems, Foster City, CA, USA). The methylation-independent *Alu* sequence was used as an endogenous control of amplification, as well as a reference for the normalization of input DNA as reported previously. A standard curve was generated using serial dilutions of bisulfite-treated CpGenome Universal Methylated DNA (Chemicon International Inc., Temecula, CA, USA). Methylation levels at each rDNAs sequence were normalized to those of CpG methylase-treated DNA. The details of all PCR primers and probes used in this assay and PCR conditions have been described previously [16].

### 4.5. Quantification of Altered Chromosomal Region in HCC

We analyzed allelic imbalance for quantification of altered chromosomal regions using the ABI PRISM Linkage Mapping Set Ver. 2 (Applied Biosystems, Foster City, CA, USA). The details of PCR amplification and electrophoresis were described previously [39,40]. We determined FAL, represented as the percentage of the locus showing allelic imbalance in the total informative alleles, as a representative of the extent of altered regions throughout the entire chromosome.

### 4.6. Statistics

We use Pearson’s chi-square test or Fisher’s exact test for comparison of categorical variables and the Wilcoxon rank-sum test and Student’s *t*-test for continuous variables. For categorization of tumors based on the methylation level of TSG promoters, we performed hierarchical clustering analyses and determined HCCs with carrying relatively high promoter methylation levels (Supplementary Figure S1a) [33]. Similarly, progression of genome-wide hypomethylation was denoted as significant hypomethylation through hierarchical clustering analyses using the methylation levels of three kinds of rDNAs (Supplementary Figure S1b) [16]. For the categorization of tumors based on the degree of chromosomal alteration, we also classified tumors using the median value of FAL as those with a FAL score ≥ 21% and those with a FAL score < 21%. For molecular classification based on mutations, DNA methylation events, and chromosomal alterations, we applied the corresponding analysis followed by hierarchical clustering analyses using x values and y values of two-dimensional drawings from the corresponding analyses. Survival between two groups was estimated using Kaplan–Meier analysis, and univariate parameters were analyzed with a log-rank test. All *p* values were two-sided, and *p* < 0.05 was considered to indicate statistical significance. All statistical analyses were conducted using the JMP version 9.0 software (SAS Institute Inc., Cary, NC, USA).

## 5. Conclusions

We concluded that molecular risks determined by common genetic and epigenetic alterations could predict metastatic recurrence after curative treatments, and could be a marker for considering systemic therapy for HCC patients.

## Figures and Tables

**Table 1:** Mutation of hepatocellular carcinoma (HCC) and clinicopathological backgrounds in the cohort of liver resection.

Clinicopathological Backgrounds		CTNNB1 Mutation			TP53 Mutation			TERT-*p* Mutation ^1^		
		+ ^2^	− ^3^	*p* Value	+	−	*p* Value	+	−	*p* Value
Sex	male	28	62	**0.0088**	22	68	0.2153	60	30	0.6083
	female	3	32		5	30		25	10	
Age	>60 years old	21	45	0.0546	13	53	0.5845	48	18	0.2308
	≤60 years old	10	49		14	45		37	22	
HBV ^4^	positive	8	21	0.6918	11	18	**0.0147**	16	13	0.0911
	negative	23	73		16	81		69	27	
HCV ^5^	positive	22	55	0.2162	15	62	0.4658	61	16	**0.0007**
	negative	9	39		12	36		24	24	
NBNC ^6^	yes	2	19	0.0755	3	18	0.3719	10	11	**0.0282**
	no	29	75		24	80		75	29	
Serum AFP level	≥200 ng/mL	4	42	**0.0015**	16	30	**0.0063**	27	19	0.0888
	<200 ng/mL	27	52		11	69		58	21	
Tumor size	≥3.0 cm	24	62	0.2607	21	65	0.2831	61	25	0.2532
	<3.0 cm	7	31		6	32		23	15	
Vascular invasion	presence	9	49	**0.0194**	19	39	**0.0029**	37	21	0.4097
	absence	22	43		7	58		46	19	
Tumor number	solitary	17	38	0.3067	10	45	0.5765	39	16	0.8219
	multiple	13	45		13	45		40	18	
Differentiation	well	16	20	**0.0018**	3	33	**0.0235**	28	8	0.1354
	moderately/poorly	15	71		23	63		55	31	

Numbers of the patients in each category and *p* values by Pearson’s chi-square test are shown. *p* values of <0.05 are shown in bold. Percentages of the patients who showed significant association between clinical backgrounds and mutations are as follows; percentage of the patients with *CTNNB1* mutation, 31% of the male vs. 8.6% of the female, 8.7% with serum AFP ≥ 200 vs. 34% with serum AFP < 200, 16% with vascular invasion vs. 34% without vascular invasion, and 44% with well-differentiated HCC vs. 17% with moderately/poorly HCC, respectively. Percentage of the patients with *TP53* mutation, 38% with HBV-positive vs. 17% with HBV-negative, 35% with serum AFP ≥ 200 vs. 14% with serum AFP < 200, 33% with vascular invasion vs. 11% without vascular invasion, and 8.3% with well-differentiated HCC vs. 27% with moderately/poorly HCC. Similarly, 79% of HCV-positive vs. 50% with HCV-negative, and 48% of NBNC vs. 72% of virus-positive are positive for TRET promoter mutation, respectively. ^1^ TERT-*p*: TERT promoter. ^2^ +: presence of mutation. ^3^ −: absence of mutation. ^4^ HBV: hepatitis B virus (positive for HBsAg), ^5^ HCV: hepatitis C virus (positive for HCVAb), ^6^ NBNC: non-B non-C (negative for both HBsAg and HCVAb).

**Table 2:** Methylation status and chromosomal alterations of HCC and clinicopathological backgrounds in the cohort of liver resection.

Clinicopathological Backgrounds		Hypermethylation of Tumor Suppressor Gene ^1^			Significant Global Hypomethylation ^2^			FAL (%) ^3^		
		+ ^2^	− ^3^	*p* Value	+	−	*p* Value	+	−	*p* Value
Sex	male	61	29	0.2636	51	39	0.2703	47	43	0.6204
	female	20	15		16	19		20	15	
Age	>60 years old	43	23	0.9306	32	34	0.2252	34	32	0.6211
	≤60 years old	38	21		35	24		33	26	
HBV ^4^	positive	17	12	0.4266	20	9	0.0583	19	10	0.1420
	negative	64	32		47	49		48	48	
HCV ^5^	positive	55	22	**0.0494**	36	41	0.0519	38	39	0.2276
	negative	26	22		31	17		29	19	
NBNC ^6^	yes	11	10	0.1914	13	8	0.4028	12	9	0.7212
	no	70	34		54	50		55	49	
Serum AFP level	≥200 ng/mL	30	16	0.9406	30	16	**0.0469**	38	8	**<0.0001**
	<200 ng/mL	51	28		37	42		29	50	
Tumor size	≥3.0 cm	58	28	0.3057	48	28	0.3849	49	37	0.2079
	<3.0 cm	22	16		18	20		17	21	
Vascular invasion	presence	40	18	0.3004	37	21	**0.0216**	39	19	**0.0043**
	absence	39	26		28	37		27	38	
Tumor number	solitary	38	17	0.3321	28	27	0.7871	30	25	0.6622
	multiple	35	23		31	27		34	24	
Differentiation	well	24	12	0.7748	18	18	0.6386	14	22	**0.0393**
	moderately/poorly	55	31		47	39		51	35	

Numbers of the patients in each category and *p* values by Pearson’s chi-square test are shown. *p* values < 0.05 are shown in bold. Percentages of the patients who showed significant association between clinical backgrounds and methylation status and chromosomal alteration are as follows; Percentage of the patients who show hypermethylation on tumor suppresser genes (TSGs), 71% of the patients with HCV-positive vs. 54% with HCV-negative, respectively. Percentage of the patients who show significant global hypomethylation, 64% with AFP ≥ 200 vs. 47% with AFP < 200, and 64% with vascular invasion vs. 43% without vascular invasion. Percentage of the patients with FAL score ≥ 21%, 83% with AFP ≥ 200 vs. 39% with AFP < 200, 67% with vascular invasion vs. 42% without vascular invasion, and 39% with well-differentiated vs. 59% with moderately/poorly-differentiated. ^1^ Methylation status in the promoters of TSGs was classified as presence (+) or absence (−) of hypermethylation, based on the cluster from hierarchal clustering analyses using methylation levels of 8 TSG promoters (*APC*, *CDKN2A*, *RASSF1A*, *HIC-1*, *GSTP1*, *RUNX3*, *SOCS1*, and *PRDM2*). ^2^ Global methylation status was classified as presence (+) or absence (−) of significant hypomethylation through hierarchal clustering analyses using methylation levels of *LINE-1*, *Alu*, and *SAT2*. ^3^ Extension of chromosomal alteration was classified using fractional allelic loss (FAL) score as FAL ≥ 21% and <21%. ^4^ HBV: hepatitis B virus (positive for HBsAg), ^5^ HCV: hepatitis C virus (positive for HCVAb), ^6^ NBNC: non-B non-C (negative for both HBsAg and HCVAb).

**Table 3:** Classification of HCCs based on the molecular alterations and clinical feature.

Characteristics of Backgrounds	A1 (%) ^1^	A2 (%)	B1 (%)	B2 (%)	*p* Value
	(*n* = 27)	(*n* = 21)	(*n* = 41)	(*n* = 36)	
***Molecular events***					
*CTNNB1* mutation					
positive (*n* = 31)	1 (4)	0 (0)	13 (32)	17 (47)	**<0.0001**
negative (*n* = 94)	26	21	28	19	
*TP53* mutation					
positive (*n* = 27)	3 (11)	0 (0)	23 (56)	1 (3)	**<0.0001**
negative (*n* = 98)	24	21	18	35	
*TERT* promoter mutation					
positive (*n* = 85)	5 (19)	15 (71)	31 (76)	34 (94)	**<0.0001**
negative (*n* = 40)	22	6	10	2	
TSG promoter hypermethylation					
presence (*n* = 81)	6 (22)	2 (10)	39 (95)	34 (94)	**<0.0001**
absence (*n* = 44)	21	19	2	2	
Significant global hypomethylation					
presence (*n* = 67)	16 (59)	2 (10)	38 (93)	11 (31)	**<0.0001**
absence (*n* = 58)	11	19	3	25	
Chromosomal alterations					
FAL ≥ 21% (*n* = 67)	19 (70)	3 (14)	37 (90)	8 (22)	**<0.0001**
FAL < 21% (*n* = 58)	8	18	4	28	
***Clinicopathological backgrounds***					
Age (years old)					
≤60 (*n* = 59)	16	7	21	15	0.2720
>60 (*n* = 66)	11	14	20	21	
Sex					
Male (*n* = 90)	20	12	30	28	0.3496
Female (*n* = 35)	7	9	11	8	
HBsAg					
Positive (*n* = 29)	10	2	12	5	0.0525
Negative (*n* = 96)	17	19	29	31	
HCVAb					
Positive (*n* = 77)	10 (37)	15 (71)	25 (61)	27 (75)	**0.0149**
Negative (*n* = 48)	17	6	16	9	
NBNC					
yes (*n* = 21)	7	4	6	4	0.4478
no (*n* = 104)	20	17	35	32	
Serum AFP level (ng/mL)					
≥200 (*n* = 46)	14 (52)	4 (19)	23 (56)	5 (14)	**0.0002**
<200 (*n* = 79)	13	17	18	31	
Tumor size (cm)					
≥3.0 (*n* = 86)	18	12	30	26	0.5117
<3.0 (*n* = 38)	9	9	10	10	
Vascular invasion					
Presence (*n* = 58)	17 (63)	5 (24)	25 (64)	11 (31)	**0.0013**
Absence (*n* = 65)	10	16	14	25	
Number of tumors					
Multiple (*n* = 58)	14	10	19	15	0.7273
Solitary (*n* = 55)	10	8	18	19	
Differentiation					
Moderately/poorly (*n* = 86)	22	12	31	21	0.0820
Well (*n* = 36)	5	8	8	15	

Numbers of the patients in each category and *p* values by Pearson’s chi-square test are shown. *p* values of <0.05 are shown in bold. TSG: tumor suppressor genes. FAL: fractional allelic loss. ^1^ Percentage of the patients with molecular alterations and each clinical feature; the percentage is shown only for the factors that show significant associations.

## Supplementary Materials

The following are available online at http://www.mdpi.com/2072-6694/10/10/367/s1, Figure S1: Classifications of HCCs based on methylation status of 8 tumor suppressor gene (TSG) promoters and 3 kinds of repetitive DNA sequences (rDNAs), Figure S2: Recurrence-free survivals of the patients in each molecular subclass. Table S1: Mutations detected in HCCs from hepatic resection, Table S2: The details of the clinical background of 25 patients who underwent liver transplantation, Table S3: Mutations detected in HCCs from liver transplantation, Table S4: Factors associated with disease-free survival of HCC patients who underwent liver transplantation, Table S5: Clinical background and molecular status of HCC cases from TCGA dataset, Table S6: The details of the clinical background of 125 patients who underwent hepatectomy, Table S7: The details of PCR primers and conditions for mutational analyses.

## References

[1] Forner A., Llovet J.M., Bruix J. (2012). Hepatocellular carcinoma. Lancet.

[2] Fernandez-Sevilla E., Allard M.A., Selten J., Golse N., Vibert E., Sa Cunha A., Cherqui D., Castaing D., Adam R. (2017). Recurrence of hepatocellular carcinoma after liver transplantation: Is there a place for resection?. Liver Transpl..

[3] Llovet J.M., Ricci S., Mazzaferro V., Hilgard P., Gane E., Blanc J.F., de Oliveira A.C., Santoro A., Raoul J.L., Forner A. (2008). Sorafenib in advanced hepatocellular carcinoma. N. Engl. J. Med..

[4] Bruix J., Qin S., Merle P., Granito A., Huang Y.H., Bodoky G., Pracht M., Yokosuka O., Rosmorduc O., Breder V. (2017). Regorafenib for patients with hepatocellular carcinoma who progressed on sorafenib treatment (RESORCE): A randomised, double-blind, placebo-controlled, phase 3 trial. Lancet.

[5] Kudo M. (2017). A New Era of Systemic Therapy for Hepatocellular Carcinoma with Regorafenib and Lenvatinib. Liver Cancer.

[6] Kudo M., Finn R.S., Qin S., Han K.H., Ikeda K., Piscaglia F., Baron A., Park J.W., Han G., Jassem J. (2018). Lenvatinib versus sorafenib in first-line treatment of patients with unresectable hepatocellular carcinoma: A randomised phase 3 non-inferiority trial. Lancet.

[7] Zhu A.X., Baron A.D., Malfertheiner P., Kudo M., Kawazoe S., Pezet D., Weissinger F., Brandi G., Barone C.A., Okusaka T. (2016). Ramucirumab as Second-Line Treatment in Patients with Advanced Hepatocellular Carcinoma: Analysis of REACH Trial Results by Child-Pugh Score. JAMA Oncol..

[8] Abou-Alfa G.K., Meyer T., Cheng A.L., El-Khoueiry A.B., Rimassa L., Ryoo B.Y., Cicin I., Merle P., Chen Y., Park J.W. (2018). Cabozantinib in Patients with Advanced and Progressing Hepatocellular Carcinoma. N. Engl. J. Med..

[9] El-Khoueiry A.B., Sangro B., Yau T., Crocenzi T.S., Kudo M., Hsu C., Kim T.Y., Choo S.P., Trojan J., Welling T.H.R. (2017). Nivolumab in patients with advanced hepatocellular carcinoma (CheckMate 040): An open-label, non-comparative, phase 1/2 dose escalation and expansion trial. Lancet.

[10] Zhu A.X., Finn R.S., Edeline J., Cattan S., Ogasawara S., Palmer D., Verslype C., Zagonel V., Fartoux L., Vogel A. (2018). Pembrolizumab in patients with advanced hepatocellular carcinoma previously treated with sorafenib (KEYNOTE-224): A non-randomised, open-label phase 2 trial. Lancet Oncol..

[11] Nishida N., Kudo M. (2018). Immune checkpoint blockade for the treatment of human hepatocellular carcinoma. Hepatol. Res..

[12] European Association for the Study of the Liver (2018). EASL Clinical Practice Guidelines: Management of hepatocellular carcinoma. J. Hepatol..

[13] Thorgeirsson S.S., Grisham J.W. (2002). Molecular pathogenesis of human hepatocellular carcinoma. Nat. Genet..

[14] Nishida N., Fukuda Y., Komeda T., Ito T., Nishimura T., Minata M., Kuno M., Katsuma H., Ikai I., Yamaoka Y. (2002). Prognostic impact of multiple allelic losses on metastatic recurrence in hepatocellular carcinoma after curative resection. Oncology.

[15] Nishida N., Nishimura T., Ito T., Komeda T., Fukuda Y., Nakao K. (2003). Chromosomal instability and human hepatocarcinogenesis. Histol. Histopathol..

[16] Nishida N., Kudo M., Nishimura T., Arizumi T., Takita M., Kitai S., Yada N., Hagiwara S., Inoue T., Minami Y. (2013). Unique association between global DNA hypomethylation and chromosomal alterations in human hepatocellular carcinoma. PLoS ONE.

[17] Totoki Y., Tatsuno K., Covington K.R., Ueda H., Creighton C.J., Kato M., Tsuji S., Donehower L.A., Slagle B.L., Nakamura H. (2014). Trans-ancestry mutational landscape of hepatocellular carcinoma genomes. Nat. Genet..

[18] Fujimoto A., Furuta M., Totoki Y., Tsunoda T., Kato M., Shiraishi Y., Tanaka H., Taniguchi H., Kawakami Y., Ueno M. (2016). Whole-genome mutational landscape and characterization of noncoding and structural mutations in liver cancer. Nat. Genet..

[19] Nishida N., Kudo M., Nagasaka T., Ikai I., Goel A. (2012). Characteristic patterns of altered DNA methylation predict emergence of human hepatocellular carcinoma. Hepatology.

[20] Nishida N., Chishina H., Arizumi T., Takita M., Kitai S., Yada N., Hagiwara S., Inoue T., Minami Y., Ueshima K. (2014). Identification of epigenetically inactivated genes in human hepatocellular carcinoma by integrative analyses of methylation profiling and pharmacological unmasking. Dig. Dis..

[21] Nishida N., Kudo M. (2014). Alteration of Epigenetic Profile in Human Hepatocellular Carcinoma and Its Clinical Implications. Liver Cancer.

[22] Nishida N., Nishimura T., Nakai T., Chishina H., Arizumi T., Takita M., Kitai S., Yada N., Hagiwara S., Inoue T. (2014). Genome-wide profiling of DNA methylation and tumor progression in human hepatocellular carcinoma. Dig. Dis..

[23] Nishida N., Iwanishi M., Minami T., Chishina H., Arizumi T., Takita M., Kitai S., Yada N., Ida H., Hagiwara S. (2015). Hepatic DNA Methylation Is Affected by Hepatocellular Carcinoma Risk in Patients with and without Hepatitis Virus. Dig. Dis..

[24] Nishida N., Nishimura T., Nagasaka T., Ikai I., Goel A., Boland C.R. (2007). Extensive methylation is associated with beta-catenin mutations in hepatocellular carcinoma: Evidence for two distinct pathways of human hepatocarcinogenesis. Cancer Res..

[25] Nishida N., Goel A. (2011). Genetic and epigenetic signatures in human hepatocellular carcinoma: A systematic review. Curr. Genom..

[26] Nishida N., Kudo M. (2016). Clinical Significance of Epigenetic Alterations in Human Hepatocellular Carcinoma and Its Association with Genetic Mutations. Dig. Dis..

[27] Hoshida Y., Toffanin S., Lachenmayer A., Villanueva A., Minguez B., Llovet J.M. (2010). Molecular classification and novel targets in hepatocellular carcinoma: Recent advancements. Semin. Liver Dis..

[28] Nishida N., Fukuda Y., Kokuryu H., Toguchida J., Yandell D.W., Ikenega M., Imura H., Ishizaki K. (1993). Role and mutational heterogeneity of the p53 gene in hepatocellular carcinoma. Cancer Res..

[29] Rebouissou S., Couchy G., Libbrecht L., Balabaud C., Imbeaud S., Auffray C., Roskams T., Bioulac-Sage P., Zucman-Rossi J. (2008). The beta-catenin pathway is activated in focal nodular hyperplasia but not in cirrhotic FNH-like nodules. J. Hepatol..

[30] Rebouissou S., Franconi A., Calderaro J., Letouze E., Imbeaud S., Pilati C., Nault J.C., Couchy G., Laurent A., Balabaud C. (2016). Genotype-phenotype correlation of CTNNB1 mutations reveals different ss-catenin activity associated with liver tumor progression. Hepatology.

[31] Desert R., Rohart F., Canal F., Sicard M., Desille M., Renaud S., Turlin B., Bellaud P., Perret C., Clement B. (2017). Human hepatocellular carcinomas with a periportal phenotype have the lowest potential for early recurrence after curative resection. Hepatology.

[32] Boyault S., Rickman D.S., de Reynies A., Balabaud C., Rebouissou S., Jeannot E., Herault A., Saric J., Belghiti J., Franco D. (2007). Transcriptome classification of HCC is related to gene alterations and to new therapeutic targets. Hepatology.

[33] Nishida N., Nagasaka T., Nishimura T., Ikai I., Boland C.R., Goel A. (2008). Aberrant methylation of multiple tumor suppressor genes in aging liver, chronic hepatitis, and hepatocellular carcinoma. Hepatology.

[34] Nault J.C., Calderaro J., Di Tommaso L., Balabaud C., Zafrani E.S., Bioulac-Sage P., Roncalli M., Zucman-Rossi J. (2014). Telomerase reverse transcriptase promoter mutation is an early somatic genetic alteration in the transformation of premalignant nodules in hepatocellular carcinoma on cirrhosis. Hepatology.

[35] Chiang D.Y., Villanueva A., Hoshida Y., Peix J., Newell P., Minguez B., LeBlanc A.C., Donovan D.J., Thung S.N., Sole M. (2008). Focal gains of VEGFA and molecular classification of hepatocellular carcinoma. Cancer Res..

[36] Schulze K., Imbeaud S., Letouze E., Alexandrov L.B., Calderaro J., Rebouissou S., Couchy G., Meiller C., Shinde J., Soysouvanh F. (2015). Exome sequencing of hepatocellular carcinomas identifies new mutational signatures and potential therapeutic targets. Nat. Genet..

[37] Gentilini D., Scala S., Gaudenzi G., Garagnani P., Capri M., Cescon M., Grazi G.L., Bacalini M.G., Pisoni S., Dicitore A. (2017). Epigenome-wide association study in hepatocellular carcinoma: Identification of stochastic epigenetic mutations through an innovative statistical approach. Oncotarget.

[38] Nault J.C., Mallet M., Pilati C., Calderaro J., Bioulac-Sage P., Laurent C., Laurent A., Cherqui D., Balabaud C., Zucman-Rossi J. (2013). High frequency of telomerase reverse-transcriptase promoter somatic mutations in hepatocellular carcinoma and preneoplastic lesions. Nat. Commun..

[39] Nishimura T., Nishida N., Itoh T., Kuno M., Minata M., Komeda T., Fukuda Y., Ikai I., Yamaoka Y., Nakao K. (2002). Comprehensive allelotyping of well-differentiated human hepatocellular carcinoma with semiquantitative determination of chromosomal gain or loss. Genes Chromosomes Cancer.

[40] Nishimura T., Nishida N., Itoh T., Komeda T., Fukuda Y., Ikai I., Yamaoka Y., Nakao K. (2005). Discrete breakpoint mapping and shortest region of overlap of chromosome arm 1q gain and 1p loss in human hepatocellular carcinoma detected by semiquantitative microsatellite analysis. Genes Chromosomes Cancer.

