# Supplementary material for: Molecular Scoring of Hepatocellular Carcinoma for Predicting Metastatic Recurrence and Requirements of Systemic Chemotherapy
Source: Cancers (Basel). 2018 Sep 29;10(10):367. doi: 10.3390/cancers10100367 (PMC6210853; doi:10.3390/cancers10100367)
Supplement: Supplementary file 1 [file cancers-10-00367-s001.zip › cancers-359971-suppl-proof-.pdf]

Supplementary Materials: Molecular Scoring of Hepatocellular Carcinoma for Predicting Metastatic Recurrence and Requirements of Systemic Chemotherapy

Naoshi Nishida, Takafumi Nishimura, Toshimi Kaido, Kosuke Minaga, Kentaro Yamao, Ken Kamata, Mamoru Takenaka, Hiroshi Ida, Satoru Hagiwara, Yasunori Minami, Toshiharu Sakurai, Tomohiro Watanabe and Masatoshi Kudo

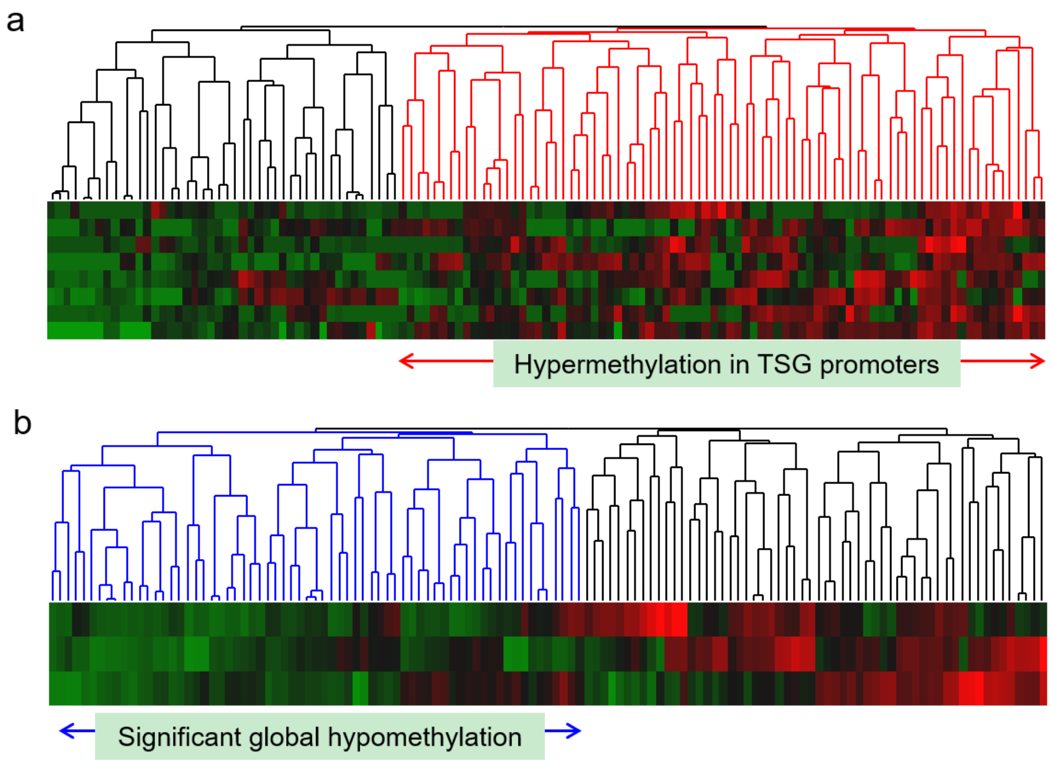

Figure S1. Classifications of HCCs based on methylation status of 8 tumor suppressor gene (TSG) promoters and 3 kind of repetitive DNA sequences (rDNAs). (**a**) HCCs with hypermethylation in the promoters of TSGs were determined using hierarchical clustering analysis. (**b**) Hypomethylation at rDNAs, which were the representative of global hypomethylation, was also determined as carrying significant hypomethylation.

Table S1. Mutations detected in HCCs from hepatic resection.

Sample ID	CNTTB1	TP53	TERT Promoter
1T	none	none	(−146) G→A
2T	none	none	(−124) G→A
3T	none	none	(−124) G→A
4T	none	none	none
5T	none	none	(−124) G→A
6T	codon 45 TCT→CCT	codon 151 CCC→GCC	(−124) G→A
7T	none	none	(−124) G→A
8T	codon 32 GAC→TAC	none	(−124) G→A
9T	codon 45 TCT→CCT	none	(−124) G→A
10T	codon 36 CAT→CGT	none	none
11T	none	none	none
12T	none	intron 7 AGgt→AGgg	(−124) G→A
13T	codon 33 TCT→TGT	none	none

14T	none	none	(−124) G→A
15T	codon 36 CAT→CCT	none	(−124) G→A
16T	none	none	none
17T	none	codon 285 GAG→TAG	(−124) G→A
18T	none	none	(−124) G→A
19T	none	none	(−124) G→A
20T	codon 41 ACC→GCC	codon 141 TGC→TAC	(−124) G→A
21T	none	codon 130 CTC→TTC	none
22T	codon 41 ACC→GCC	none	(−124) G→A
23T	codon 45 TCT→TTT	none	(−124) G→A
24T	none	none	(−124) G→A
25T	none	none	(−124) G→A
26T	none	intron 6 AGgt→AGgg	(−124) G→A
27T	none	codon 208 GAC→GCC	(−124) G→A
28T	none	none	(−124) G→A
29T	none	none	(−124) G→A
30T	none	codon 247 CGG→CAG	none
31T	none	codon 278 CCT→TCT	(−124) G→A
32T	none	none	(−124) G→A
33T	none	none	(−124) G→A
34T	none	none	none
35T	none	none	(−124) G→A
36T	codon 45 TCT→CCT	none	(−124) G→A
37T	none	codon 147 del (1bp)	none
38T	none	none	none
39T	codon33 TCT→CCT	none	(−124) G→A
40T	none	none	(−124) G→A
41T	none	none	(−124) G→A
42T	codon37 TCT→TGT	codon 193 CAT→TAT	(−124) G→A
43T	none	none	(−124) G→A
44T	none	none	(−124) G→A
45T	none	none	none
46T	none	codon 161 GCC→TCC	(−146) G→A
47T	codon33 TCT→TTT	none	(−124) G→A
48T	none	none	none
49T	none	none	none
50T	codon44 CCT→GCT	codon 177 CCC→CGC	(−124) G→A
51T	none	codon 132 AAG→AAT	(−124) G→A
52T	none	none	(−124) G→A
53T	none	none	(−124) G→A
54T	codon32 GAC→AAC	none	(−124) G→A
55T	none	intron 5 agGT→atGT	(−124) G→A
56T	none	none	(−124) G→A
57T	none	none	none
58T	codon 36 CAT→CCT	codon 215 AGT→AAT	(−124) G→A
59T	none	codon 139 AAG→ACG	none
60T	none	codon 157 GTC→GCC	(−124) G→A
61T	none	none	none
62T	none	intron 4 tcctacagTA→ccctacagTA	(−124) G→A
63T	none	codon 244 GGC→TGC	(−124) G→A
64T	none	codon 156 CGC→CAC	none
65T	none	codon 240 AGT→CGT	(−124) G→A
66T	none	none	none
67T	none	none	(−124) G→A
68T	codon34 GGA→AGA	none	(−127) G→A<br>(−124) G→A
69T	none	none	(−124) G→A
70T	none	none	none
71T	none	none	none
72T	none	none	none
73T	none	codon 258 GAA→AAA	none
74T	codon34 GGA→GTA	none	none
75T	none	none	none
76T	codon45 TCT→TTT	codon 285 GAG→AAG	none
77T	none	codon 236 TAC→TGC	(−124) G→A
78T	none	none	(−124) G→A

79T	none	none	(−124) G→A
80T	none	none	(−124) G→A
81T	none	none	none
82T	none	none	none
83T	none	none	(−124) G→A
84T	codon 44 to 55 del (33bp)	none	(−124) G→A (−68) G→A
85T	codon 37 TCT→TTT	none	(−124) G→A
86T	codon 45 TCT→CCT	none	(−124) G→A
87T	none	none	(−124) G→A
88T	none	none	(−124) G→A
89T	codon 41 ACC→GCC	none	(−124) G→A
90T	codon 41 ACC→GCC	none	(−124) G→A
91T	none	none	(−124) G→A
92T	codon 45 TCT→CCT	none	none
93T	none	none	none
94T	none	none	(−124) G→A
95T	codon 41 ACC→GCC	codon 239 AAC→GAC	(−124) G→A
96T	codon 41 ACC→GCC	none	(−124) G→A
97T	none	none	(−124) G→A
98T	none	none	none
99T	codon36 CAT→CCT	none	(−124) G→A
100T	none	none	none
101T	none	none	none
102T	none	none	(−124) G→A
103T	none	none	(−124) G→A
104T	none	none	none
105T	none	none	(−124) G→A
106T	none	none	(−124) G→A
107T	none	none	(−124) G→A
108T	none	none	(−124) G→A
109T	codon 32 GAC→AAC	none	(−124) G→A
110T	codon 45 TCT→CCT	none	none
111T	none	none	(−124) G→A
112T	none	none	none
113T	codon 41 ACC→GCC	none	(−124) G→A
114T	none	none	(−124) G→A
115T	none	none	(−124) G→A
116T	none	none	(−124) G→A
117T	none	codon 270 TTT→GTT	none
118T	none	none	(−124) G→A
119T	none	none	none
120T	none	none	(−124) G→A
121T	none	none	(−124) G→A
122T	none	none	none
123T	none	none	none
124T	none	none	(−124) G→A
125T	none	none	none

Table S2. The details of the clinical background of 25 patients who underwent liver transplantation.

Clinical Factors	Median (25th–75th Percentile) or Number of Cases
Age (years old)	59 (54–62.5)
Sex	
Male/female	19/6
Virus	
HBV/HCV/HBV& HCV/negative	10/13/1/1
Serum AFP [1] (ng/mL)	41 (9.4–222)
Serum DCP [2] (mAU/mL)	54 (16.8–151.5)
Maximum tumor size (cm)	2.6 (1.8–3.2)
Number of tumor	
Single/multiple/missing	3/22
Tumor differentiation	
Well/moderately/poorly/missing	3/17/5

[1] AFP; α-fetoprotein [2] Des-gamma carboxyprothrombin.

Table S3. Mutations detected in HCCs from liver transplantation.

Sample ID	CNTTB1	TP53	TERT promoter
T03H	none	none	(−124) G→A
T04H	none	none	none
T05H	codon 41 ACC→GCC	codon 183 TCA→CCA	none
T10H	none	codon 181 CGC→TGC	(−124) G→A
T15H12	none	none	(−124) G→A
T21H1	none	codon 237 ATG→ATA	(−124) G→A
T22H	codon 45 TCT→CCT	none	(−124) G→A
T26H1	none	codon 286 GAA→TAA	none
T29H	none	codon 234 TAC→TAA	(−124) G→A
T32H	none	none	(−124) G→A
T35H1	none	none	(−124) G→A
T38H1	none	none	none
T42H	none	none	(−124) G→A
T43H	none	none	none
T47H	codon 45 to 47 del (9 bp)	none	(−124) G→A
T51H	codon 32 GAC→GGC	none	(−124) G→A
T52H2	none	none	none
T53H	none	none	(−124) G→A
T55H	none	codon 239 AAC→GAC	(−124) G→A
T57H1	codon 45 TCT→TTT	codon 248 CGG→TGG	(−124) G→A
T61H4	codon 32 GAC→GGC	none	(−124) G→A
T77H1	none	none	(−124) G→A
T78H1	none	codon 173 GTG→ATG	none
T86H1	none	intron 5 TGgt→TGgc	(−124) G→A
T95H2	codon 41 ACC→GCC	none	(−124) G→A

Table S4. Factors associated with disease-free survival of HCC patients who underwent liver transplantation.

**Clinicopathological backgrounds**	**No. of Cases**		**Univariate**	**Multivariate**	
	**Total**	**With Event**	***p* value**	***p* value**	**HR (95% CI)**
AFP					
≥200 ng/mL	6	5	<0.0001	0.0969	14.8 (0.64–1413)
<200 ng/mL	18	1			1
PIVKA-II					
≥400 AU/mL	3	3	0.0003	0.5050	2.01 (0.27–22.6)
<400 AU/mL	21	3			1
Size					
≥3.0 cm	9	5	0.0170	0.9617	0.93 (0.04–31.0)
<3.0 cm	16	1			
Differentiation					
Mod-poor	22	6	0.3325		
Well	3	0			
Number of tumor					
Multiple	22	6	0.3325		
Single	3	0			
Milan criteria					
Out	17	6	0.1782		
In	8	0			
Molecular pattern					
Aggressive	10	5	0.0090	0.8200	1.42 (0.07–44.0)
Mild	14	1			

supplementary S5 in an individual.

Table S6. The details of the clinical background of 125 patients who underwent hepatectomy.

Clinical Factors	Median (25th–75th Percentile) or Number of Cases
Age (years old)	63 (56–69)
Sex (male/female)	90/35
Virus	
HBV/HCV/HBV& HCV/negative	27/75/2/21
Serum AFP [1)](ng/mL)	55 (7.3–487.5)
Maximum tumor size (cm)	3.6 (2.7–6)
Vascular invasion	
Presence/absence/missing	58/65/2
Number of tumor	
Single/multiple/missing	55/58/12
Tumor differentiation	
Well/moderately/poorly/missing	36/64/21/4

[1)] AFP; α fetoprotein.

Table S7. The details of PCR primers and conditions for mutational analyses.

Target genes	Primers	Tm
CNTTB1 exon 3	Forward: 5′-(ATGGAACCAGACAGAAAAG)-3′<br>Reverse: 5′-(TACAGGACTTGGGAGGTATC)-3′	58 °C
TP53 exon 5-1	Forward: 5′-(TTATCTGTTCACTTGTGCCC)-3′<br>Reverse: 5′-(TCATGTGCTGTGACTGCTTG)-3′	60 °C
TP53 exon 5-2	Forward: 5′-(TTCCACACCCCCGCCCGGCA)-3′<br>Reverse: 5′-(ACCCTGGGCAACCAGCCCTG)-3′	65 °C
TP53 exon 6	Forward: 5′-(ACGACAGGGCTGGTTGCCCA)-3′<br>Reverse: 5′-(CTCCCAGAGACCCCAGTTGC)-3′	65 °C
TP53 exon 7	Forward: 5′-(GGCCTCATCTTGGGCCTGTG)-3′<br>Reverse: 5′-(CAGTGTGCAGGGTGGCAAGT)-3′	64 °C
TP53 exon 8	Forward: 5′-(CTGCCTCTTGCTTCTCTTTT)-3′<br>Reverse: 5′-(TCTCCTCCACCGCTTCTTGT)-3′	60 °C
TERT promoter	Forward: 5′-(CAGCGCTGCCTGAAACTC)-3′<br>Reverse: 5′-(GTCCTGCCCCTTCACCTT)-3′	62 °C

© 2018 by the authors. Licensee MDPI, Basel, Switzerland. This article is an open access article distributed under the terms and conditions of the Creative Commons Attribution (CC BY) license (http://creativecommons.org/licenses/by/4.0/).
